# Psychosocial burdens in early- versus late-onset dementia: analysis of discrimination, stress, and loneliness in the All of Us Research Program

**DOI:** 10.1093/geroni/igaf087

**Published:** 2025-08-07

**Authors:** Xiang Qi, Zhiyue Mo, Junyu Sui, Yanping Jiang, Bei Wu

**Affiliations:** Rory Meyers College of Nursing, New York University, New York, New York, United States; Department of Applied Statistics, Social Science, and the Humanities, New York University, New York, New York, United States; Rory Meyers College of Nursing, New York University, New York, New York, United States; Edson College of Nursing and Health Innovation, Arizona State University, Phoenix, Arizona, United States; Department of Family Medicine and Community Health, Institute for Health, Health Care Policy and Aging Research, Rutgers University, New Brunswick, New Jersey, United States; Rory Meyers College of Nursing, New York University, New York, New York, United States

**Keywords:** Alzheimer’s disease, Mental health, Psychology of aging/psychiatry, Young-onset dementia

## Abstract

**Background and Objectives:**

Early-onset dementia (EOD, onset before age 65) is relatively rare but often devastating for patients and families. Individuals with dementia face stigma and psychosocial burdens; however, it is unclear whether those with EOD experience worse psychosocial outcomes than those with late-onset dementia (LOD) or no dementia. This study examined differences in psychosocial outcomes across EOD, LOD, and no-dementia groups.

**Research Design and Methods:**

This cross-sectional study used data from the All of Us Research Program surveys and linked electronic health records (EHR). Diagnosis of dementia was identified through electronic health records (EOD [*n* = 442], LOD [*n* = 658], and without dementia [*n* = 79,035]). Outcomes included everyday discrimination, discrimination in healthcare settings, perceived stress, and loneliness. Negative binomial regression models were employed to compare outcomes by dementia status, adjusting for demographic, socioeconomic, and health-related covariates.

**Results:**

EOD participants reported the highest mean levels of all psychosocial outcomes (e.g., everyday discrimination score of 8.3 in EOD vs 4.6 in LOD and 6.8 in no-dementia). In the fully-adjusted models, EOD was associated with significantly higher everyday discrimination (incidence rate ratio [IRR] = 1.30, 95% CI 1.05–1.62), discrimination in healthcare settings (IRR = 1.08, 95% CI 1.01–1.15), and perceived stress (IRR = 1.09, 95% CI 1.02–1.15) compared with LOD. No difference in loneliness was observed between EOD and LOD (IRR = 1.03, 95% CI 0.98–1.09). Compared with those without dementia, the EOD group also showed elevated levels of all outcomes. All differences remained significant after adjusting for covariates.

**Discussion and Implications:**

Findings highlight the unique challenges faced by young adults with EOD and underscore the need for targeted interventions to reduce psychosocial burden in this growing population. As the prevalence of EOD continues to rise, clinicians and policymakers should prioritize supportive resources to mitigate these disparities for EOD patients and their families.

Translational Significance Individuals with early-onset dementia (EOD) face unique psychosocial burdens, including discrimination, stress, and loneliness. Our study found that people with EOD reported significantly higher levels of everyday discrimination, discrimination in healthcare settings, and perceived stress compared with those with late-onset dementia or no dementia. These findings highlight the need for targeted efforts across individual, healthcare, and societal levels to reduce stigma and psychosocial burdens. By informing public health initiatives and guiding improvements in clinical care, this research can enhance well-being and help transform healthcare delivery and societal support systems for people with EOD.

Dementia is a progressive neurocognitive disorder characterized by a decline in memory, thinking, and daily functioning. The Alzheimer’s Association (2024) report estimates that about 6.9 million Americans aged 65 and older are living with dementia (Alzheimer’s Association, 2024). There are two major subtypes of dementia categorized by age of onset: early-onset dementia (EOD) and the more common late-onset dementia (LOD). EOD (also known as young-onset dementia) occurs before age 65 (McMurtray et al., 2006), and, while relatively uncommon (around 200,000 people in the United States (Hendriks et al., 2021), it poses a rising public health problem associated with severe stress and burden on patients and their families. Globally, the burden of EOD has increased significantly over the past few decades and is projected to continue rising, reaching over 10 million cases by 2050 (Feng et al., 2025). Compared with LOD, EOD is often marked by a more rapid progression, earlier emergence of symptoms in mid-life, and a greater disruption to the patient’s and family’s quality of life (Chang et al., 2017; McMurtray et al., 2006).

Dementia is also heavily stigmatized in society, leading to marginalization and discrimination against those affected (Henderson & Thornicroft, 2009). This stigma stems from a lack of public awareness and understanding about the disease, as well as negative stereotypes and prejudices (Blake & Hopper, 2022; Kim et al., 2019). Studies have found that even healthcare professionals can hold stigmatizing attitudes toward individuals with dementia (Aminzadeh et al., 2012; Gove et al., 2016). For instance, stereotypes that portray individuals with dementia as dangerous or incompetent may lead healthcare providers to unconsciously devalue the autonomy and capabilities of these patients (Nguyen & Li, 2020). Such stigma-fueled behaviors may lead to patients feeling devalued and can cause miscommunication, misdiagnosis, and psychological distress. Overall, the diagnosis of dementia often subjects individuals to mistreatment and bias in everyday interactions (Barnes et al., 2012).

Individuals with EOD may face double stigma—the combined stigma of dementia and being younger than typical for this condition. Because they do not fit the expected age profile for dementia, EOD patients can be misunderstood or socially ostracized by peers and even healthcare providers, who might treat them as anomalously “old” or incapable (Issakainen et al., 2023). EOD often disrupts individuals’ careers and family life in mid-adulthood, which can further exacerbate feelings of marginalization and discrimination (Velilla et al., 2022). However, the exact extent and nature of discrimination faced by people with EOD remain unclear, warranting further investigation. In addition to discrimination, individuals with dementia frequently experience heightened loneliness and stress. Prior studies have found that people with dementia report greater overall, emotional, and social loneliness compared with those without dementia (Lee et al., 2022; Moyle et al., 2011). High levels of chronic stress are also reported; for example, one study noted that EOD is linked to higher life stress compared with LOD (Novek et al., 2016). Furthermore, individuals with dementia have markedly increased risks of psychiatric disorders and use of psychotropic medications both before and after their dementia diagnosis (Mo et al., 2023), indicating substantial psychological strain. Despite these insights, it remains unknown how younger people with dementia compare with older people with dementia in terms of both perceived stress and loneliness. In particular, whether EOD individuals experience disproportionate levels of stress and loneliness, beyond what is observed in LOD, has not been well established.

To address these knowledge gaps, we utilized data from the All of Us Research Program (AoU) (All of Us Research Program Investigators et al., 2019). In this study, we examined four key psychosocial outcomes: everyday discrimination, discrimination in healthcare settings, perceived stress, and loneliness. Our analysis compared these outcomes across three groups—individuals with EOD, those with LOD, and those with no dementia—to clarify the psychosocial profile of EOD. We formulated two hypotheses:

Hypothesis 1: Individuals with EOD would experience higher levels of everyday discrimination, discrimination in healthcare settings, perceived stress, and loneliness compared with those with LOD.

Hypothesis 2: Individuals with EOD would also report higher levels of these outcomes compared with individuals without dementia.

## Method

### Study population

**Figure 1.**
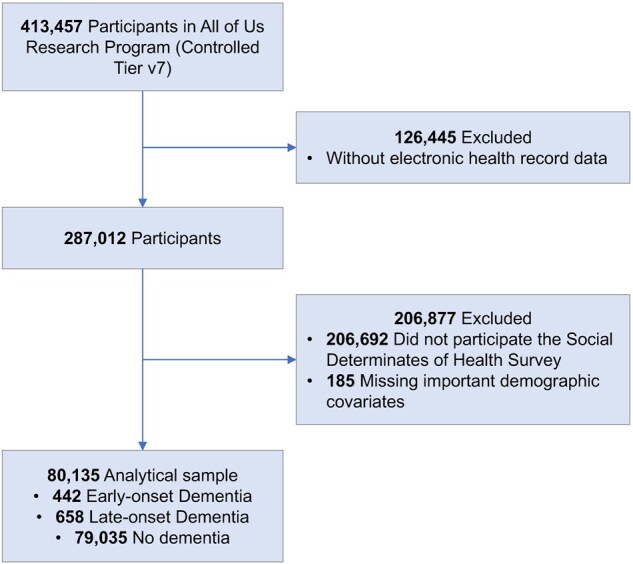
Sample selection flowchart in All of Us Research Program.

The study used data from the AoU Research Program, a National Institutes of Health project that aims to recruit over one million diverse individuals across the United States to advance precision medicine (All of Us Research Program Investigators et al., 2019). AoU provides a large, population-based database containing survey data, genomic analyses, longitudinal electronic health records (EHR), physical measurements, and more from adult participants to study factors influencing health and disease. Enrollment began in May 2018 and is open to adults aged 18 or older from over 340 recruitment sites nationwide. At the time of our analysis (controlled tier v7 data, July 2022), 413,457 participants were enrolled in AoU. Among them, 287,012 provided their EHRs. Participants complete a series of surveys, including those assessing social determinants of health and psychosocial factors, at enrollment or during ­follow-up. For this study, we included all AoU participants with available data on at least one of the four psychosocial outcomes of interest described below. We identified a total of 80,135 participants with complete data for the variables of interest (see Figure 1 for cohort flow). The study population comprised 442 individuals with EOD, 658 with LOD, and 79,035 individuals without any dementia diagnosis. The AoU institutional review board approved all study procedures, and all participants provided informed consent (including consent for researchers to access their EHR data). Our analysis was conducted in accordance with the AoU data user code of conduct.

### Measurements

#### Exposure: dementia status

The exposure was dementia status, categorized as EOD, LOD, or no dementia. Dementia diagnoses were ascertained from EHR data using International Classification of Diseases (ICD-9/10) codes indicative of Alzheimer’s disease and related dementias (see Supplementary Table 1 [see online supplementary material] for code list). Participants whose first documented dementia diagnosis occurred before age 65 were classified as having EOD (Li et al., 2023; McMurtray et al., 2006). Participants whose first dementia diagnosis was at age 65 or older were classified as LOD. All other participants with no recorded dementia diagnosis in their EHRs comprised the no dementia group.

#### Outcomes: psychosocial measures

We focused on four common psychosocial measurement scales: the Everyday Discrimination Scale (Williams et al., 1997), the Discrimination Scale in Healthcare Settings (Hausmann et al., 2010), the Cohen Perceived Stress Scale (Cohen et al., 1983), and the Short-form UCLA Loneliness scale (Hughes et al., 2004). Everyday discrimination experiences were measured by the 9-item Everyday Discrimination Scale, with total scores ranging from 0 to 45 (higher scores indicate more frequent experiences of discrimination). Discrimination in healthcare settings was assessed with a 7-item scale (scores 7 to 35, higher scores reflect greater perceived discrimination in healthcare settings). Perceived stress was evaluated using the 10-item Perceived Stress Scale (scores 10 to 50; higher scores indicate higher stress). Loneliness was measured with the 8-item short-form UCLA Loneliness Scale (scores 8 to 32, higher scores indicate greater loneliness). These psychosocial instruments have been widely utilized in older adult populations (Aravena et al., 2024; Bregola et al., 2022; Dark et al., 2023; Jiang et al., 2017), including individuals with cognitive impairment (Aravena et al., 2024; Bregola et al., 2022; Dark et al., 2023; Ros et al., 2011), supporting their suitability for use among people with mild-to-moderate dementia. Furthermore, all four scales demonstrated strong internal consistency in our analytic sample (Cronbach’s α = 0.80–0.92), indicating reliable measurement. Sample items and reliability for each scale are provided in Table 1.

**Table 1.** Measurement outlines for four psychosocial outcomes.

Measurements	Items, scale	Total scores	Example items	Cronbach’s α
Everyday Discrimination Scale	9-item, 0–5 scale	0–45, higher total scores indicate more frequent experiences of discrimination	“You are treated with less courtesy than other people” “You are treated with less respect than other people are”	0.92
Discrimination Scale in Healthcare Settings	7-item, 1–5 scale	7–35, higher total scores indicate a greater level of perceived discrimination experienced by the patient in healthcare settings	“You feel like a doctor or nurse is not listening to what you were saying” “A doctor or nurse acts as if he or she thinks you are not smart”	0.89
Perceived Stress Scale	10-item, 1–5 scale	10–50, higher scores indicate higher perceived stress	“In the last month, how often have you felt that you were unable to control the important things in your life?”	0.80
Short-form UCLA Loneliness Scale	8-item, 1–4 scale	8–32, higher scores indicate greater loneliness	“I feel isolated from other” “There is no one I can turn to”	0.86

#### Covariates

Based on prior research on psychosocial well-being in dementia (Bannon et al., 2022; Lee et al., 2022), we included a range of demographic, socioeconomic, and health-related covariates. Demographic covariates were: age at survey completion (categorized as 18–44, 45–59, 60–74, or ≥75 years), gender (woman, man, or other/non-binary including transgender, etc.), and race/ethnicity (Non-Hispanic White, Non-Hispanic Black, Hispanic, or Other [including Asian, Native Hawaiian/Pacific Islander, Middle Eastern/North African, and any other/mixed backgrounds]). Socioeconomic covariates included annual household income (<$50,000; $50,000–$100,000; >$100,000; or unknown), educational attainment (high school or less; college degree or higher; or unknown), health insurance status (insured vs uninsured), marital status (married/partnered vs not married), nativity (US-born vs not US-born), living ­arrangement (living alone vs with others), and neighborhood deprivation index (a composite measure of neighborhood socioeconomic disadvantage based on American Community Survey data (Knighton et al., 2016), range 0 to 1 with higher values indicating more deprivation). Health-related covariates derived from EHRs included indicators for three common chronic conditions (hypertension, obesity, and type 2 diabetes), and three measures of functional ability limitations (self-­reported difficulty climbing stairs, difficulty dressing or bathing, and difficulty running errands alone, each coded as yes or no). Missing data in covariates were relatively minimal; any missing values were coded as “unknown” category to retain those participants in adjusted analyses. We opted to adjust for these specific prevalent chronic conditions rather than employing a comprehensive comorbidity index (e.g., Charlson Comorbidity Index) to maintain parsimony and reduce potential multicollinearity; these three conditions were selected for their relevance and high prevalence. In addition, we did not include depressive symptoms as a covariate. While depressive symptoms are strongly associated with stress, loneliness, and discrimination, they may lie on the causal pathway or represent part of the psychosocial burden of dementia. Thus, adjusting for depressive symptoms could have obscured the true relationships of interest, so we chose to exclude depressive symptoms from our models.

#### Statistical analysis

We first performed descriptive analyses to characterize the study population by dementia status. For continuous variables (e.g., neighborhood deprivation), we computed mean values and standard deviations, and we tested differences across the three groups using analysis of variance (ANOVA). For categorical variables (e.g., gender, income level), we tabulated frequencies and percentages and used Chi-square tests to compare distributions by group.

Next, we determined the appropriate modeling approach for each of the four outcome variables. Since the discrimination, stress, and loneliness scores are essentially count or index variables with skewed distributions, we compared three candidate distributions for regression modeling: Poisson, Gamma, and Negative Binomial. Goodness-of-fit was evaluated via log-­likelihood, information criteria, and visual diagnostics (density plots, Q–Q plots, etc.; see Supplementary Figures 1–8, see online supplementary material). The Negative Binomial distribution provided the best fit for all four outcomes (overdispersion was present, making Poisson less suitable). Therefore, we proceeded with negative binomial regression models to ­estimate associations between dementia status and each outcome.

Our primary regression analyses included the dementia status groups (with LOD or no dementia as reference categories, as described below) and adjusted sequentially for covariates. We fitted three models for each comparison: Model 1 unadjusted, Model 2 adjusted for demographic and socioeconomic factors, and Model 3 fully adjusted (including chronic conditions and functional ability covariates). All analyses were conducted using R version 4.3.2 within the AoU Researcher Workbench. Two-sided *p*-values < .05 were considered statistically significant.

## Results

### Characteristics of the study population

Table 2 presents a summary of the sample characteristics by dementia status. Among 80,135 AoU participants with complete data (442 with EOD, 658 with LOD, and 79,035 with no dementia), we observed notable demographic and health differences by dementia status. The mean age of EOD participants was 60.1 years (by definition, all <65 at diagnosis), whereas those with LOD had a mean age of 77.3 years, and the no-dementia group had a mean age of 52.9 years. The EOD group had significantly lower income and education levels and a lower proportion of married individuals compared with both the LOD and non-dementia groups (all *p* < .001). In contrast, EOD participants exhibited higher rates of obesity, type 2 diabetes, and functional impairments (e.g., difficulties in climbing stairs or running errands) than the other groups.

**Table 2.** Descriptive characteristics by dementia status (*N* = 80,315).

Variable	Early-onset dementia (*n* = 442)	Late-onset dementia (*n* = 658)	No dementia (*n* = 79,035)	*p* value
Age, No. (%)				<.001
18–44	44 (9.95)	0	17,567 (22.2)	
45–59	129 (29.2)	0	18,742 (23.7)	
60–74	245 (55.4)	307 (46.7)	32,562 (41.2)	
75+	24 (5.43)	351 (53.3)	10,164 (12.9)	
Gender, No. (%)				<.001
Women	266 (60.2)	350 (53.2)	49,776 (63.0)	
Man	166 (37.6)	291 (44.2)	26,798 (33.9)	
Other^a^	10 (2.26)	17 (2.58)	2,461 (3.11)	
Race/ethnicity, No. (%)				<.001
White	304 (68.8)	531 (80.7)	59,317 (75.1)	
Black	51 (11.5)	39 (5.93)	6,265 (7.93)	
Hispanic	35 (7.92)	26 (3.95)	5,497 (6.96)	
Other^b^	52 (11.8)	62 (9.42)	7,956 (10.1)	
Income level, No. (%)				<.001
<$50,000	195 (44.1)	201 (30.5)	21,948 (27.8)	
$50,000–$100,000	83 (18.8)	188 (28.6)	20,862 (26.4)	
>$100,000	91 (20.6)	149 (22.6)	26,459 (33.5)	
Unknown	73 (16.5)	120 (18.2)	9,766 (12.4)	
Educational attainment, No. (%)				<.001
College or higher	351 (79.4)	555 (84.3)	67,708 (85.7)	
High school or less	82 (18.6)	85 (12.9)	9,268 (11.7)	
Unknown	9 (2.04)	18 (2.74)	2,059 (2.61)	
Marital status, No. (%)				<.001
Married	200 (45.2)	392 (59.6)	48,120 (60.9)	
Not married	230 (52.0)	244 (37.1)	28,798 (36.4)	
Unknown	12 (2.71)	22 (3.34)	2,117 (2.68)	
Nativity, No. (%)				.151
US	397 (89.8)	585 (88.9)	69,749 (88.3)	
Other	37 (8.37)	52 (7.90)	7,627 (9.65)	
Unknown	8 (1.81)	21 (3.19)	1,659 (2.10)	
Neighborhood deprivation index, *M* (*SD*)^c^	0.31 (0.06)	0.31 (0.06)	0.31 (0.06)	.006
Difficulty of climbing, No. (%)				<.001
Yes	56 (12.7)	83 (12.6)	4,553 (5.76)	
No	136 (30.8)	177 (26.9)	37,339 (47.2)	
Unknown	250 (56.6)	398 (60.5)	37,143 (47.0)	
Difficulty of dressing and bathing, No. (%)				<.001
Yes	18 (4.07)	25 (3.80)	1,243 (1.57)	
No	176 (39.8)	238 (36.2)	40,728 (51.5)	
Unknown	248 (56.1)	395 (60.0)	37,064 (46.9)	
Difficulty of running errands alone, No. (%)				<.001
Yes	29 (6.56)	42 (6.38)	2,197 (2.78)	
No	165 (37.3)	220 (33.4)	39,726 (50.3)	
Unknown	248 (56.1)	396 (60.2)	37,112 (47.0)	
Hypertension, No. (%)				<.001
Yes	307 (69.5)	523 (79.5%)	32,656 (41.3%)	
No	135 (30.5)	135 (20.5%)	46,379 (58.7%)	
Obesity, No. (%)				<.001
Yes	229 (51.8%)	264 (40.1%)	19,819 (25.1%)	
No	213 (48.2%)	394 (59.9%)	59,216 (74.9%)	
Type 2 diabetes, No. (%)				<.001
Yes	164 (37.1%)	221 (33.6%)	12,102 (15.3%)	
No	278 (62.9%)	437 (66.4%)	66,933 (84.7%)	

a “Other” gender group: includes non-binary, transgender, not man only, not woman only, prefer not to answer, skipped, or no matching concept.

b “Other” race and ethnicity group: Middle Eastern or North African descent, Native Hawaiian and Pacific Islander, Asian, none of these, skip and prefer not to answer.

c Neighborhood deprivation index ranges from 0 to 1 (a higher index indicates more neighborhood deprivation).

**Figure 2.**
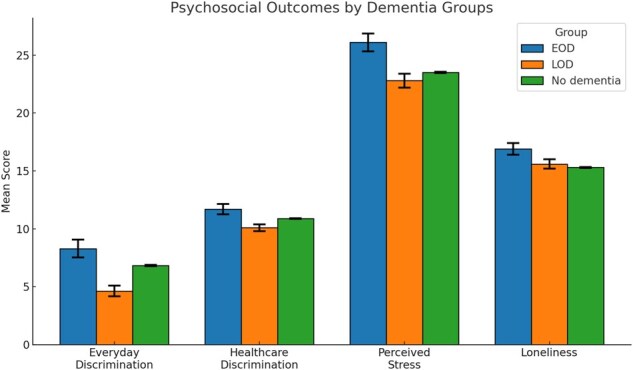
Total scores of everyday discrimination, discrimination in healthcare settings, perceived stress, and loneliness by dementia status. Early-onset dementia (EOD) participants reported higher average levels of discrimination and stress than late-onset dementia (LOD) and no-­dementia groups. The EOD group also had slightly higher loneliness than the no-dementia group. Point estimates with 95% confidence interval error bars are shown for each group.

As illustrated in Figure 2, individuals with EOD reported the highest average levels on all four psychosocial outcomes, indicating a greater psychosocial burden. Specifically, everyday discrimination (mean 8.30 vs 4.63 in LOD and 6.83 in no-­dementia), discrimination in healthcare settings (11.7 vs 10.1 in LOD and 10.9 in no-dementia), perceived stress (26.1 vs 22.8 in LOD and 23.5 in no-dementia), and loneliness (16.9 vs 15.6 in LOD and 15.3 in no-dementia) were all highest in the EOD group. To further unpack these differences, we conducted an item-level analysis of each psychosocial scale (see Supplementary Table 2, see online supplementary material). This analysis revealed that specific items—such as feeling disrespected or isolated—showed particularly large differences between EOD and LOD groups, helping to clarify the domains where disparities are most pronounced.

### Psychological outcomes: EOD vs LOD

Table 3 presents incidence rate ratio (IRRs) for the association between EOD and each outcome in three sequential models. In the unadjusted model (Model 1), EOD status was significantly associated with higher scores on everyday discrimination (IRR 1.79, 95% CI 1.51–2.12), discrimination in healthcare (IRR 1.16, 95% CI 1.10–1.22), psychological distress (IRR 1.15, 95% CI 1.10–1.20), and loneliness (IRR 1.08, 95% CI 1.04–1.13) compared with LOD. In Model 2, adjusting for demographic and socioeconomic factors attenuated these associations, suggesting that some of the difference is explained by sociodemographic characteristics. Further adjustment for chronic conditions and functional status in Model 3 resulted in IRRs that were largely unchanged from Model 2. In the fully adjusted model, individuals with EOD had an everyday discrimination score 1.30 times that of the LOD group (*p* = .018). The discrimination in healthcare settings score was 8% higher in EOD than in LOD (IRR 1.08, 95% CI 1.01–1.15, *p* = .020). The perceived stress score was 1.09 times higher in EOD than in LOD (*p* = .006). By contrast, there was no significant difference in loneliness between the EOD and LOD groups (IRR 1.03, 95% CI 0.98–1.09, *p* = .276).

**Table 3.** Multivariable analysis on the psychosocial outcomes in EOD vs no dementia and EOD vs LOD.

Outcomes	Model 1		Model 2		Model 3	
	IRR (95% CI)	*p* value	IRR (95% CI)	*p* value	IRR (95% CI)	*p* value
**EOD vs LOD (Ref.)**						
Everyday discrimination	**1.79 (1.51–2.12)**	**<.001**	**1.29 (1.04–1.60)**	**.021**	**1.30 (1.05–1.62)**	**.018**
Discrimination in medical setting	**1.16 (1.10–1.22)**	**<.001**	**1.07 (1.01–1.14)**	**.029**	**1.08 (1.01–1.15)**	**.020**
Perceived stress	**1.15 (1.10–1.20)**	**<.001**	**1.08 (1.02–1.15)**	**.007**	**1.09 (1.02–1.15)**	**.006**
Loneliness	**1.08 (1.04–1.13)**	**<.001**	1.03 (0.97–1.08)	.281	1.03 (0.98–1.09)	.276
**EOD vs no dementia (Ref.)**						
Everyday discrimination	**1.22 (1.08–1.37)**	**<.001**	**1.18 (1.05–1.32)**	**.004**	**1.13 (1.01–1.27)**	**.034**
Discrimination in medical setting	**1.08 (1.04–1.12)**	**<.001**	**1.06 (1.02–1.10)**	**<.001**	**1.04 (1.01–1.08)**	**.031**
Perceived stress	**1.11 (1.07–1.15)**	**<.001**	**1.11 (1.07-–1.14)**	**<.001**	**1.09 (1.05–1.12)**	**<.001**
Loneliness	**1.11 (1.07–1.14)**	**<.001**	**1.06 (1.03–1.09)**	**<.001**	**1.04 (1.01–1.08)**	**.009**

*Note.* Bolded statistics usually indicate results that are statistically significant (*p* < .05). Model 1 was unadjusted. Model 2 was adjusted for demographic and socioeconomic variables (age, gender, race/ethnicity, income level, education level, marital status, nativity, neighborhood deprivation index). Model 3 was additionally adjusted for health status (difficulty of climbing, difficulty of dressing and bathing, running errands alone, hypertension, obesity, type 2 diabetes). EOD = early-onset dementia; LOD = late-onset dementia; IRR = incident rate ratio; CI = confidence interval.

### Psychological outcomes: EOD vs no dementia

As presented in Table 3, when comparing EOD participants with those without dementia, we found that the EOD group scored higher on all four outcomes even after full adjustments. In the fully adjusted model (Model 3), EOD was associated with 13% higher everyday discrimination scores (*p* = .034) and 4% higher healthcare discrimination scores (*p* = .031) relative to the no-dementia group. Likewise, EOD participants had significantly greater perceived stress (9% higher) and loneliness (4% higher) compared with individuals without dementia (both *p* < .01). Although adjustment for covariates reduced the magnitude of these differences, the persistence of significant gaps suggests that EOD confers an additional psychological burden beyond what is explained by sociodemographic and health factors alone.

## Discussion

In this large, diverse cohort, we found that individuals with EOD bear a disproportionately high psychosocial burden. Not only did the EOD group report more frequent experiences of discrimination and higher perceived stress than both LOD and participants without dementia, but they also exhibited elevated loneliness compared with those without dementia. These findings advance our understanding of EOD by directly comparing it with LOD in the context of psychosocial outcomes. To our knowledge, this is the first population-based study to quantify discrimination, stress, and loneliness in EOD versus LOD, revealing a clear pattern of greater psychosocial adversity in the early-onset group.

A key finding is that people with EOD are significantly more likely to experience discrimination—both in everyday situations and in healthcare settings—relative to older people with dementia. This aligns with prior evidence that dementia-­related stigma remains pervasive and can manifest as mistreatment or marginalization of affected individuals (Bacsu et al., 2020; Low & Purwaningrum, 2020). Stigma stems from negative stereotypes that paint dementia as a hopeless, dehumanizing condition, which can lead others to treat individuals with dementia as “less than” or to exclude them socially (Blake & Hopper, 2022; Henderson & Thornicroft, 2009). In healthcare, such stigma may translate into providers communicating inadequately or bypassing the patient’s input. For instance, Canada’s national dementia strategy described scenarios where physicians were reluctant to disclose a dementia diagnosis and tended to address caregivers rather than the patients, resulting in patient disempowerment and even misdiagnoses (Bacsu et al., 2020). These discriminatory practices take a tangible toll: stigma-driven interactions with healthcare providers have been linked to poorer quality of care and psychological distress for people with dementia (Bacsu et al., 2020). Our findings suggest that these issues may be exacerbated for those with EOD, who often face a “double stigma” at the intersection of dementia and ageism. Unlike typical older patients, a person in mid-adulthood with dementia deviates from social expectations, which can prompt peers, employers, or even clinicians to view them through a lens of both cognitive impairment and “being too young” for such a condition (Issakainen et al., 2023). This dual stigmatization likely contributes to the higher discrimination scores we observed in the EOD group. Furthermore, younger adults with dementia might be more sensitive to or aware of unfair treatment, given that they are navigating work and family roles where age-related expectations are different from those of retirees (Yap et al., 2011). Previous research indicates that younger adults are more prone to avoid seeking healthcare due to anticipated stigma (Yap et al., 2011) or past negative experiences with providers, especially when structural barriers like lack of insurance are present (Leyva et al., 2020). Indeed, not being old enough to qualify for Medicare or other age-based benefits can leave many EOD patients without adequate health insurance coverage, creating a structural form of discrimination that impedes access to care (Leyva et al., 2020). Together, these factors highlight why EOD patients may encounter more frequent and impactful discrimination in multiple facets of life.

Our results also demonstrated that EOD is associated with heightened stress levels. EOD participants had higher perceived stress scores than both LOD and no-dementia groups, even after accounting for covariates. The demands of coping with dementia at an atypical age—such as abrupt disruption of one’s career, raising children while dealing with cognitive decline, or struggling with financial instability due to lost income—can all serve as potent stressors for individuals with EOD and their families (Bannon et al., 2020). Notably, we observed that the EOD group had a higher prevalence of conditions like obesity and type 2 diabetes compared with the LOD group, which may be influenced in part by chronic stress. Prolonged psychological stress triggers elevated secretion of cortisol, a stress hormone that, when persistently high, can contribute to weight gain and metabolic disturbances. Numbers of studies showed that individuals with higher work and life stress had significantly greater odds of cardiometabolic diseases (Kivimäki et al., 2023). Thus, the elevated stress among EOD patients may be one factor driving their higher rates of chronic illnesses, compounding their health challenges. These insights underscore the importance of stress management and support for mid-life individuals coping with dementia—addressing psychological distress in this group could have downstream benefits for their physical health as well.

Loneliness is another critical dimension of psychosocial burden that our study assessed. We found that both EOD and LOD patients reported markedly high levels of loneliness, far exceeding those of the general population without dementia. While the difference in loneliness between EOD and LOD was not statistically significant, EOD individuals were significantly lonelier than those without dementia. The high loneliness in EOD is especially concerning given that these individuals are in midlife, a stage when people are typically engaged in family, social, and professional networks. Qualitative studies have highlighted that younger people with dementia frequently feel isolated and misunderstood in their social spheres (Bannon et al., 2020; Popok et al., 2022). Unlike older retirees, many EOD patients lose the ability to continue working or to partake in the busy social lives their age peers enjoy. As a result, they can become disconnected from colleagues and friends, who often struggle to relate to their condition. Our findings and prior research suggest that EOD patients face a unique social vacuum: their contemporaries are not expecting to be caregivers or to accommodate cognitive impairment in a friend at such a young age, and services like dementia support groups are often geared toward much older adults (Bannon et al., 2020; Bannon et al., 2022; Popok et al., 2022). This gap can lead to EOD individuals feeling “out of place” even in settings intended for dementia support, thereby compounding their sense of loneliness. Importantly, loneliness itself can feed back into worse health outcomes; it has been linked to depression, sleep disturbance, faster cognitive decline, and poorer quality of life (Lee et al., 2022; Qi et al., 2023a, 2023b; Qiao et al., 2022; Salinas et al., 2022). Younger patients might experience a more profound mismatch between their social needs and available support (Qi et al., 2025), which calls for tailored interventions—such as peer networks or mentoring programs connecting younger dementia patients and families—to mitigate loneliness in this population.

In our study, an unexpected pattern emerged: the no-­dementia comparison group reported higher levels of everyday and healthcare discrimination and stress than the LOD group. This paradox may reflect differences in age and cognitive status influencing self-reports of psychosocial burden. Older adults with LOD might underreport discrimination and stress due to age-related positivity bias and cognitive impairment. Research shows that older people tend to recall fewer negative experiences and focus more on positive information, resulting in lower negative affect in daily life (Carstensen & DeLiema, 2018). In addition, cognitive deficits in dementia can diminish awareness of negative experiences. Many individuals with Alzheimer’s disease, for example, exhibit anosognosia—a lack of insight into their own deficits—which is common at the dementia stage (present in up to 80% of patients) (Vannini et al., 2020). This reduced awareness may extend to perceiving or remembering stressful or unfair treatment, leading LOD participants to underreport discrimination and stress. By contrast, younger or cognitively intact individuals (such as the no-dementia group) may be more exposed and attuned to discrimination and stressors. They typically engage in more diverse social and healthcare interactions and are better able to recognize subtle biases. Indeed, studies find that perceived mistreatment and everyday discrimination are often reported more frequently by younger adults than by older adults (Ariza-Montes et al., 2017; Potter et al., 2019). Thus, the no-dementia group’s higher reported discrimination and stress could stem from greater exposure to these experiences and the absence of the dampening effects of age-related positivity bias or cognitive impairment on negative perceptions.

Several limitations of our study should be acknowledged. First, the cross-sectional design limits our ability to establish causal relationships or temporal ordering between dementia onset and the psychosocial outcomes. Second, the substantial attenuation of the group differences after adjusting for demographic and socioeconomic covariates suggests that some of these factors may mediate the association between EOD status and psychosocial outcomes. We may be underestimating the true impact of EOD on discrimination, stress, and loneliness if factors like income, education, or comorbidities are themselves downstream effects of EOD. Third, genetic factors play a significant role in the development of EOD (Nudelman et al., 2023), and such familial early-onset cases might coincide with unique socioeconomic challenges. Additionally, EOD often imposes heavy caregiving and financial burdens on spouses/partners in mid-life, potentially affecting marriage and social connectedness. Thus, controlling for marital status and socioeconomic variables in our analysis might mask some of the true psychosocial impact of EOD. Fourth, there is a potential for selection bias in our sample. Participants in the AoU database who completed the required surveys and assessments may be somewhat higher-­functioning; those with very severe dementia (especially in the EOD group) could be underrepresented due to the difficulty of participating in research activities. The requirement for in-person enrollment and surveys may have excluded individuals with advanced dementia or those without caregiver support, leading our analytic sample to skew toward those with milder symptoms and better resources. Fifth, the three comparison groups differed substantially in size (the no-dementia group was much larger than the dementia groups). Although negative binomial regression is generally robust to unequal group sizes, such imbalance could still pose analytical challenges. We adjusted for a wide range of covariates to mitigate confounding, but it remains possible that the stark sample size differences may have influenced the precision of our estimates. Finally, depressive symptoms, which are known to be strongly associated with stress, loneliness, and perceptions of discrimination, were not controlled as a covariate due to conceptual overlap with all psychosocial outcomes. Future studies should examine how depressive symptoms interact with psychosocial experiences to clarify their mediating or moderating roles.

## Conclusion

Individuals with EOD experience significantly higher levels of everyday discrimination, discrimination in healthcare settings, and perceived stress compared with those with LOD, whereas loneliness levels did not significantly differ between the EOD and LOD groups. Findings highlight a profound and multifaceted psychological burden in younger people facing dementia. Given the rising prevalence of EOD as a global health concern, addressing the excess discrimination, stress, and isolation experienced by EOD patients could yield benefits not only for the individuals themselves but also for their families and care partners. By tailoring support and resources to reduce stigma, support mental well-being, and enhance social connectedness for those with EOD, we can strive to mitigate the psychosocial disparities identified in this study and promote more inclusive, compassionate care for all people living with dementia.

## Supplementary Material

igaf087_Supplementary_Data

## Data Availability

Data from this study are accessible to qualified researchers with approved controlled-tier access via the All of Us Researcher Workbench (researchallofus.org). In line with participant privacy protections and NIH All of Us Research Program policies, these data are maintained by the All of Us program and are not publicly available. The study was not preregistered prior to analysis.
