# Supplementary material for: Psychosocial burdens in early- versus late-onset dementia: analysis of discrimination, stress, and loneliness in the All of Us Research Program
Source: Innov Aging. 2025 Aug 7;9(9):igaf087. doi: 10.1093/geroni/igaf087 (PMC12505131; doi:10.1093/geroni/igaf087)

***Innovation in Aging* Supplementary Material: Qi, Mo, Sui, Jiang, & Wu.** **Psychosocial Burdens in Early- versus Late-Onset Dementia: Analysis of Discrimination, Stress, and Loneliness in the All of Us Research Program.**

**Supplementary Table 1. ICD-9-CM and ICD-10-CM diagnosis codes for dementia**

| **ICD Version** | **Code** | **Description** |
| --- | --- | --- |
| ICD9-CM | 290.0 | Senile dementia, uncomplicated |
| ICD9-CM | 290.1 | Presenile dementia |
| ICD9-CM | 290.2 | Senile dementia with delusional or depressive features |
| ICD9-CM | 290.3 | Senile demetia with delirium |
| ICD9-CM | 290.4 | Vascular dementia |
| ICD9-CM | 290.8 | Other specified senile psychotic conditions |
| ICD9-CM | 290.9 | Unspecified senile psychotic condition |
| ICD9-CM | 291.2 | Alcohol-induced persisting dementia |
| ICD9-CM | 292.82 | Drug-induced persisting dementia |
| ICD9-CM | 292.83 | Drug-induced persisting amnestic disorder |
| ICD9-CM | 294.0 | Amnestic disorder classified elsewhere (Korsakoffs) |
| ICD9-CM | 294.1 | Dementia in conditions classifed elsewhere |
| ICD9-CM | 294.2 | Dementia, unspecified |
| ICD9-CM | 294.8 | Other persistent mental disorders due to conditions classified elsewhere |
| ICD9-CM | 331.0 | Alzheimer's disease |
| ICD9-CM | 331.11 | Pick's Disease |
| ICD9-CM | 331.19 | Other frontotemporal dementia |
| ICD9-CM | 331.2 | Senile degeneration of brain |
| ICD9-CM | 331.7 | Cerebral degeneration in diseases classified elsewhere |
| ICD9-CM | 331.82 | Dementia with lewy bodies |
| ICD9-CM | 331.89 | Other cerebral degeneration |
| ICD9-CM | 331.9 | Cerebral degeneration, unspecified |
| ICD9-CM | 797 | Senility without mention of psychosis |
| ICD10-CM | F01 | Vascular dementia |
| ICD10-CM | F01.5 | Vascular dementia |
| ICD10-CM | F02 | Dementia in other diseases classified elsewhere |
| ICD10-CM | F03 | Unspecified dementia |
| ICD10-CM | F04 | Amnestic disorder due to known physiological condition |
| ICD10-CM | F10.26 | Alcohol dependence with alcohol induced persisting amnestic disorder |
| ICD10-CM | F10.27 | Alcohol dependence with alcohol induced persisting dementia |
| ICD10-CM | G30 | Alzheimer's disease |
| ICD10-CM | G31.0 | Frontotemporal dementia |
| ICD10-CM | G31.1 | Senile degeneration of brain, not elsewhere classified |
| ICD10-CM | G31.2 | Degeneration of nervous system due to alcohol |
| ICD10-CM | G31.83 | Dementia with Lewy bodies |
| ICD10-CM | G31.89 | Other specified degenerative diseases of nervous system |
| ICD10-CM | G31.9 | Degenerative disease of nervous system, unspecified |
| ICD10-CM | G32 | Other degenerative disorders of nerous system in diseases classifed elsewhere |
| ICD10-CM | G32.8 | Other specified degenerative disorders of nervous system in diseases classified elsewhere |
| ICD10-CM | G94 | Other disorders of brain in diseases classified elsewhere |

**Supplementary Table 2. Item-level mean (SD) scores by dementia groups**

| **Scale / Item** | **EOD (n=442)** | **LOD (n=658)** | **No Dementia (n=79,035)** |
| --- | --- | --- | --- |
| **Everyday Discrimination Scale (9 items)** |  |  |  |
| Treated with less courtesy than others | 1.31 (1.29) | 0.87 (0.98) | 1.09 (1.18) |
| Treated with less respect than others | 1.20 (1.19) | 0.78 (0.88) | 1.03 (1.13) |
| Receive poorer service than others (restaurants/stores) | 1.01 (1.11) | 0.63 (0.77) | 0.90 (1.02) |
| People act as if you are not smart | 1.07 (1.29) | 0.57 (0.82) | 0.75 (0.98) |
| People act as if they are afraid of you | 0.73 (1.00) | 0.27 (0.67) | 0.63 (1.08) |
| People act as if you are dishonest | 0.85 (0.88) | 0.40 (0.72) | 0.69 (1.01) |
| People act as if they’re better than you | 1.08 (1.08) | 0.77 (0.88) | 1.01 (1.20) |
| Called names or insulted | 0.62 (1.01) | 0.06 (0.29) | 0.37 (0.92) |
| Threatened or harassed | 0.49 (0.93) | 0.14 (0.33) | 0.28 (0.80) |
| **Discrimination in Healthcare Settings (7 items)** |  |  |  |
| Treated with less courtesy than others (health care) | 1.84 (1.11) | 1.63 (0.89) | 1.72 (1.02) |
| Treated with less respect than others (health care) | 1.73 (0.99) | 1.50 (0.80) | 1.60 (0.92) |
| Receive poorer service than others (health care) | 1.49 (0.98) | 1.34 (0.81) | 1.44 (0.89) |
| Doctor or nurse acts as if you are not smart | 1.91 (1.18) | 1.51 (1.02) | 1.61 (1.10) |
| Doctor or nurse acts as if he/she is afraid of you | 0.72 (0.79) | 0.55 (0.60) | 0.71 (0.78) |
| Doctor or nurse acts as if he/she is better than you | 1.87 (1.10) | 1.69 (1.02) | 1.81 (0.97) |
| Doctor/nurse is not listening to you | 2.21 (0.93) | 1.91 (1.00) | 2.10 (0.90) |
| **Perceived Stress Scale (10 items)** |  |  |  |
| Upset by unexpected events (last month) | 2.65 (1.08) | 2.35 (0.91) | 2.52 (0.98) |
| Felt unable to control important things | 2.38 (1.00) | 1.81 (0.99) | 1.92 (1.11) |
| Felt nervous and “stressed” | 2.75 (0.90) | 2.49 (0.80) | 2.59 (0.91) |
| Felt confident about handling personal problems ***(reverse-scored)*** | 1.08 (0.78) | 1.61 (0.89) | 1.50 (0.91) |
| Felt things were going your way ***(reverse-scored)*** | 1.34 (0.93) | 1.72 (1.00) | 1.61 (0.97) |
| Could not cope with all the things you had to do | 2.37 (1.09) | 2.24 (0.90) | 2.32 (0.99) |
| Able to control irritations in your life ***(reverse-scored)*** | 1.22 (0.77) | 1.62 (0.92) | 1.54 (0.92) |
| Felt on top of things ***(reverse-scored)*** | 1.29 (0.72) | 1.49 (0.77) | 1.44 (0.79) |
| Angered by things outside of your control | 2.33 (1.12) | 1.87 (0.97) | 2.03 (1.08) |
| Felt difficulties were piling up too high | 2.38 (0.99) | 2.15 (1.00) | 2.24 (0.99) |
| **UCLA Loneliness Scale (8 items)** |  |  |  |
| I lack companionship | 2.38 (1.08) | 2.40 (1.17) | 2.27 (1.28) |
| There is no one I can turn to | 1.89 (1.01) | 1.91 (1.21) | 1.80 (1.09) |
| I feel left out | 2.47 (1.01) | 2.34 (1.07) | 2.32 (1.22) |
| I feel isolated from others | 2.48 (0.99) | 2.35 (1.10) | 2.38 (1.18) |
| I am unhappy being so withdrawn | 2.18 (0.99) | 2.03 (1.01) | 2.00 (1.10) |
| People are around me but not with me | 2.57 (1.08) | 2.54 (1.20) | 2.45 (1.29) |
| I am an outgoing person ***(reverse-scored)*** | 1.45 (1.10) | 1.86 (1.20) | 1.99 (1.40) |
| I can find companionship when I want it ***(reverse-scored)*** | 1.70 (1.12) | 1.96 (1.21) | 1.95 (1.33) |

**Note: All values represent mean (SD) on the original item response scales.**

**Supplementary Figure 1. Model selection of Everyday Discrimination Scale, comparing EOD to no dementia**

**Supplementary Figure 2. Model selection of Everyday Discrimination Scale, comparing EOD to LOD**


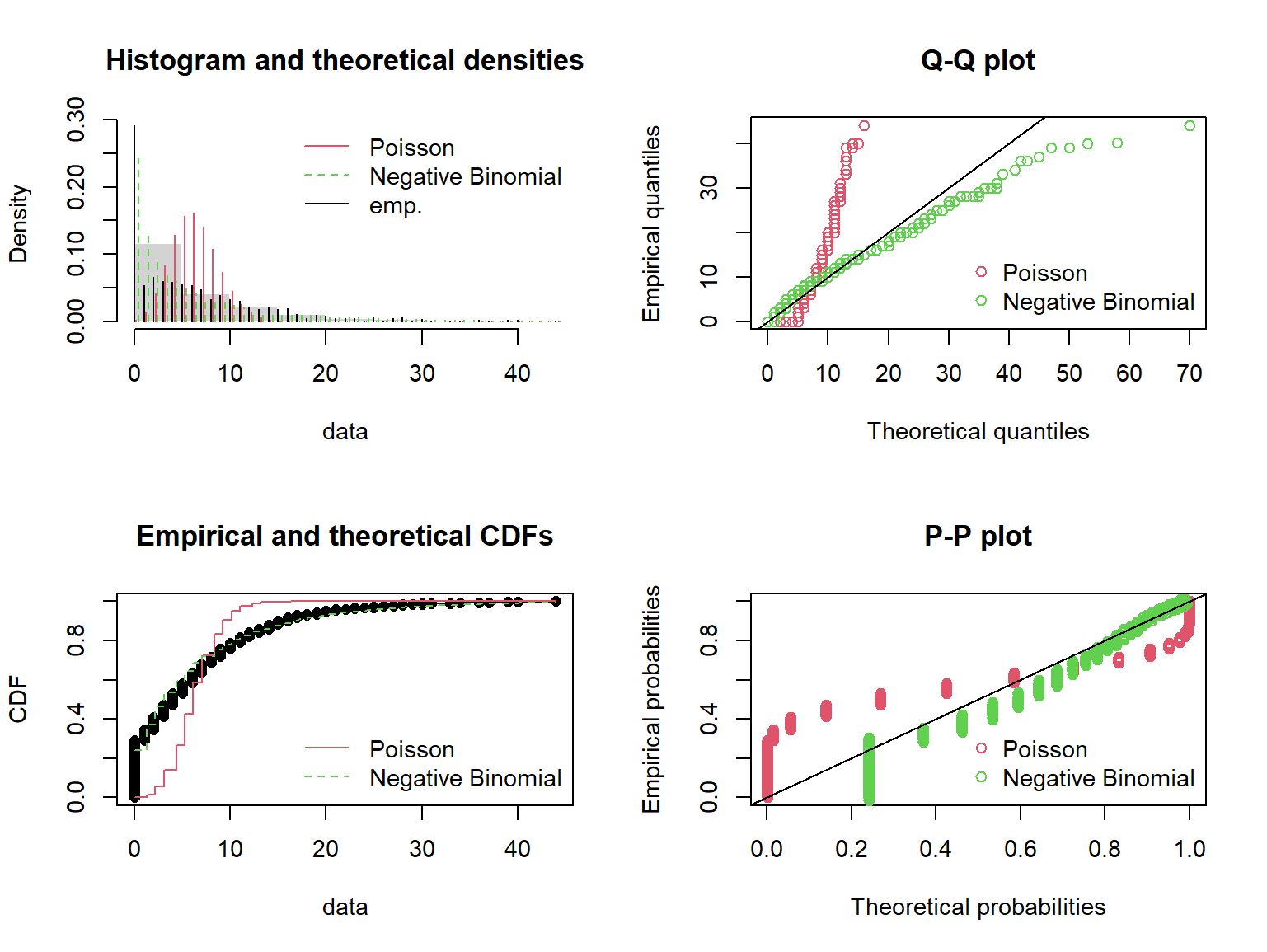


**Supplementary Figure 3. Model selection of Discrimination in Medical Setting Scale, comparing EOD to no dementia**


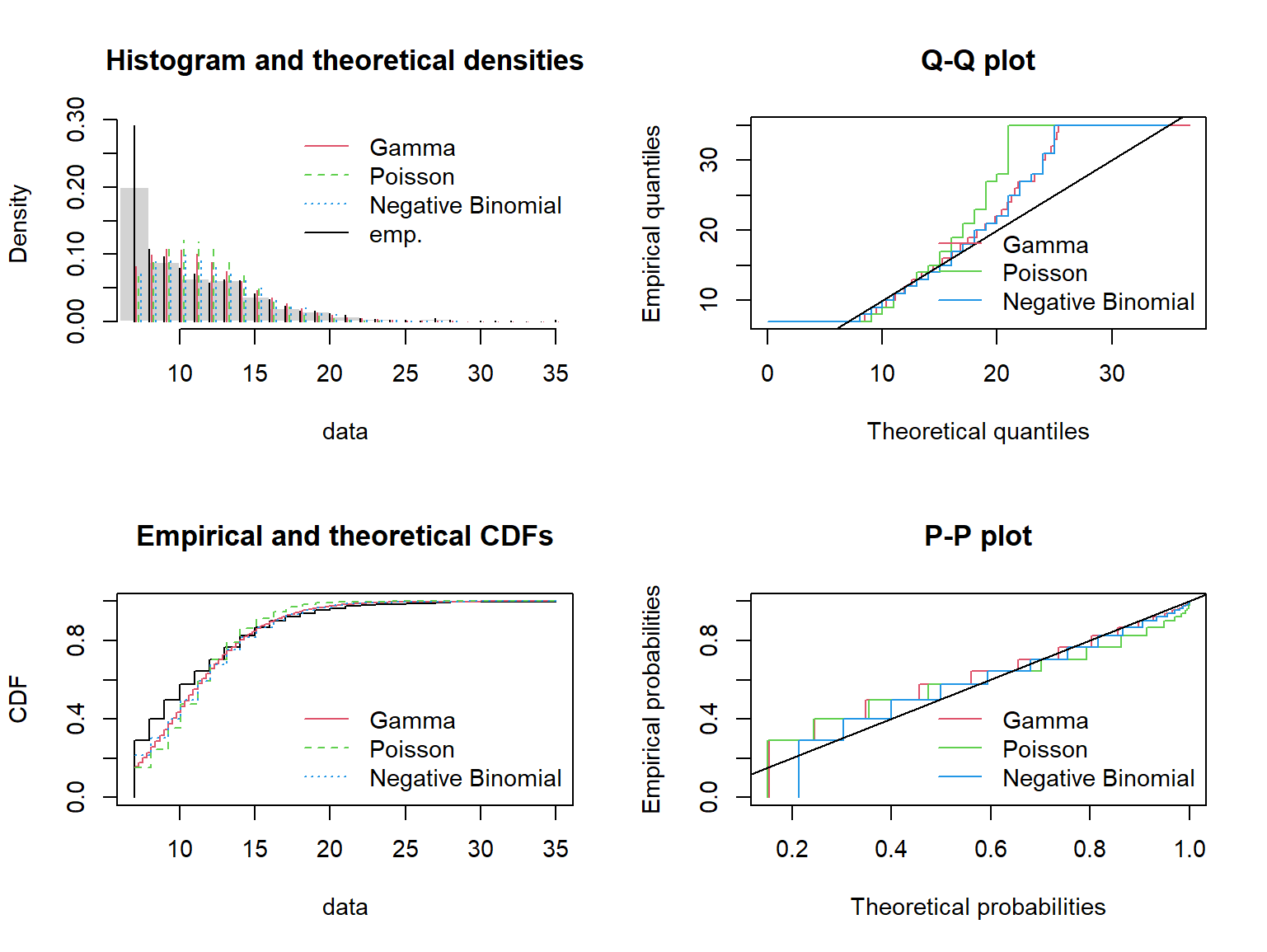


**Supplementary Figure 4. Model selection of discrimination in medical setting scale, comparing EOD to LOD**


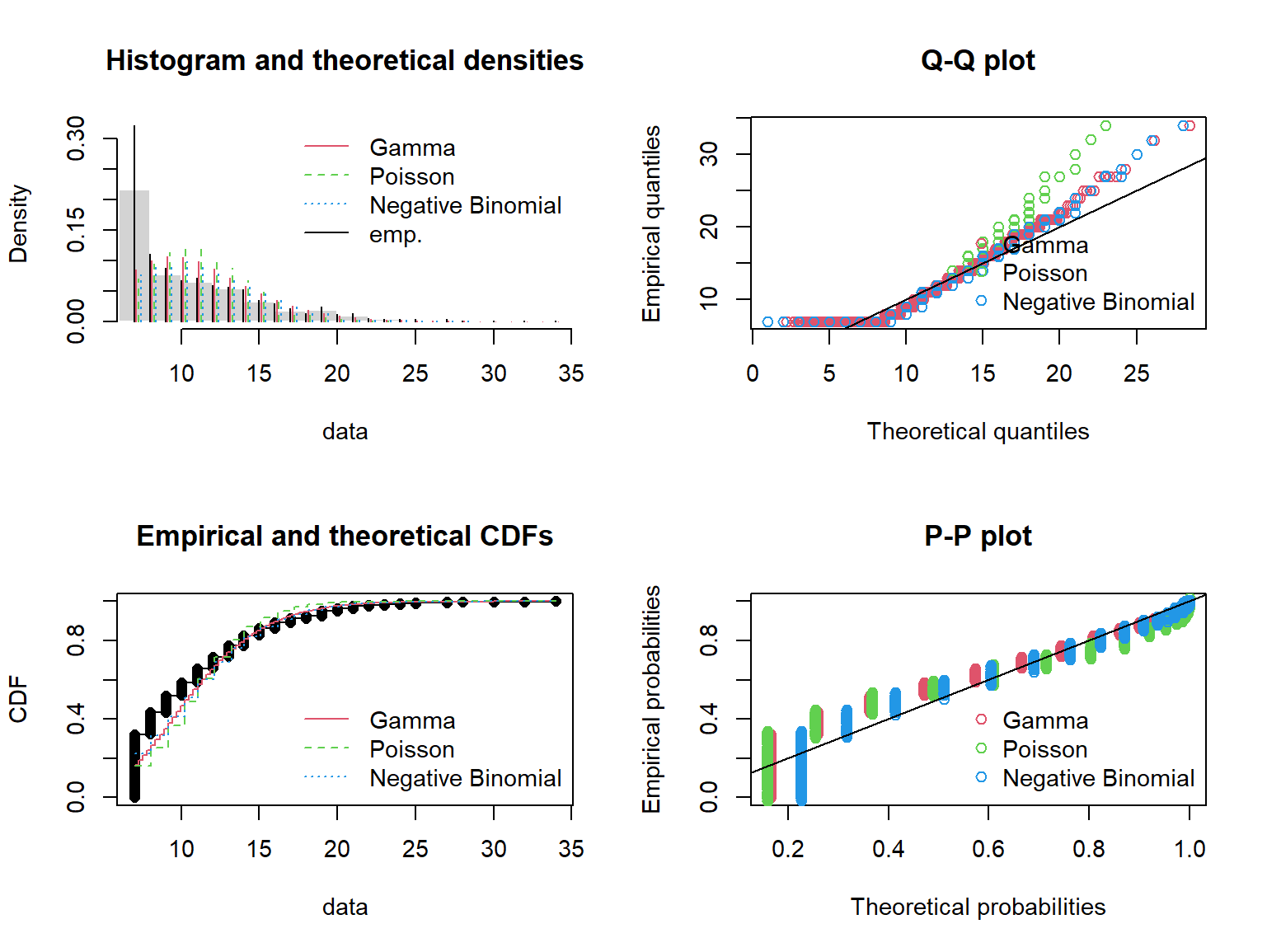


**Supplementary Figure 5. Model selection of Perceived Stress Scale, comparing EOD to no dementia**


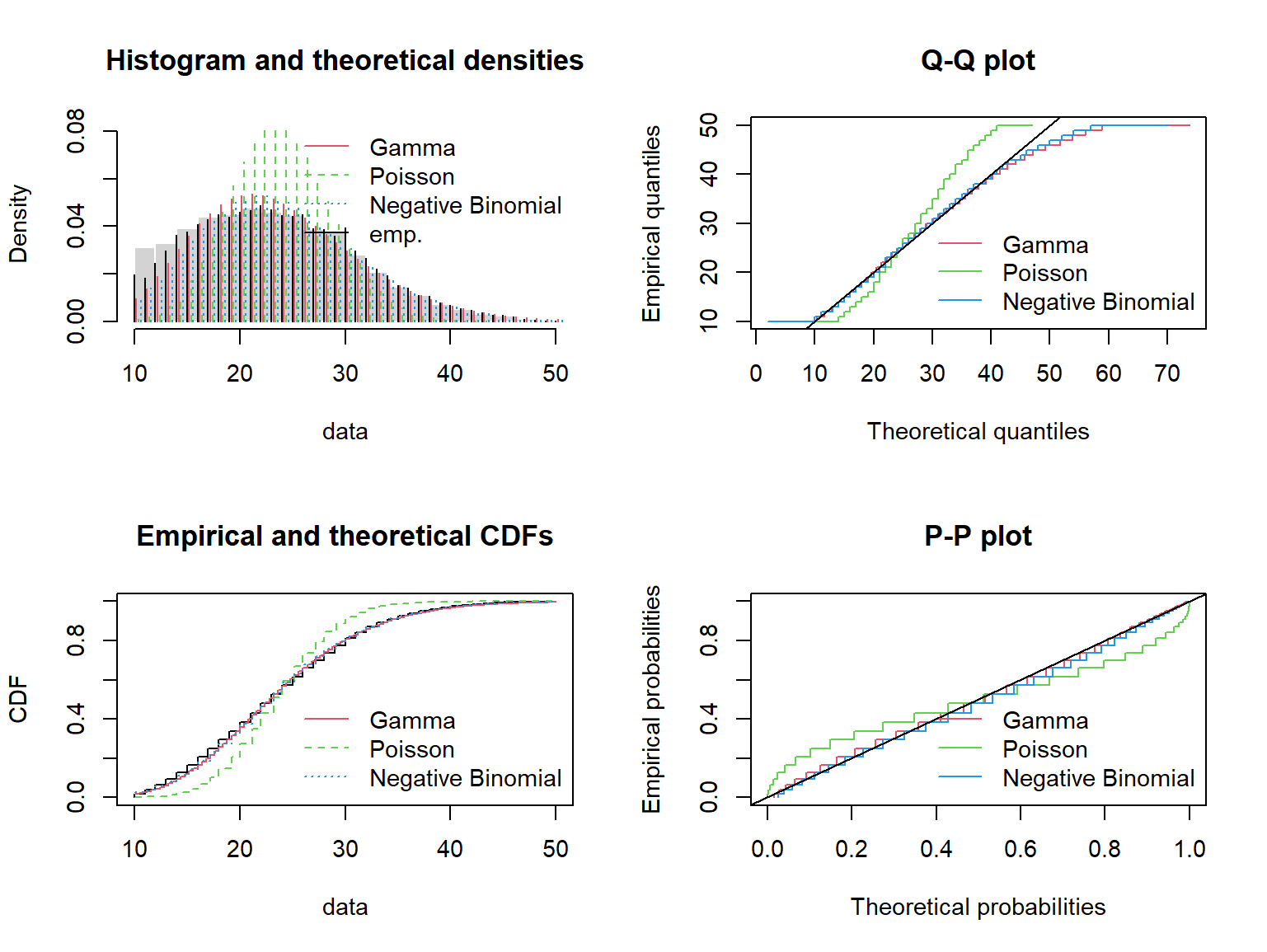


**Supplementary Figure 6. Model selection of Perceived Stress Scale, comparing EOD to LOD**


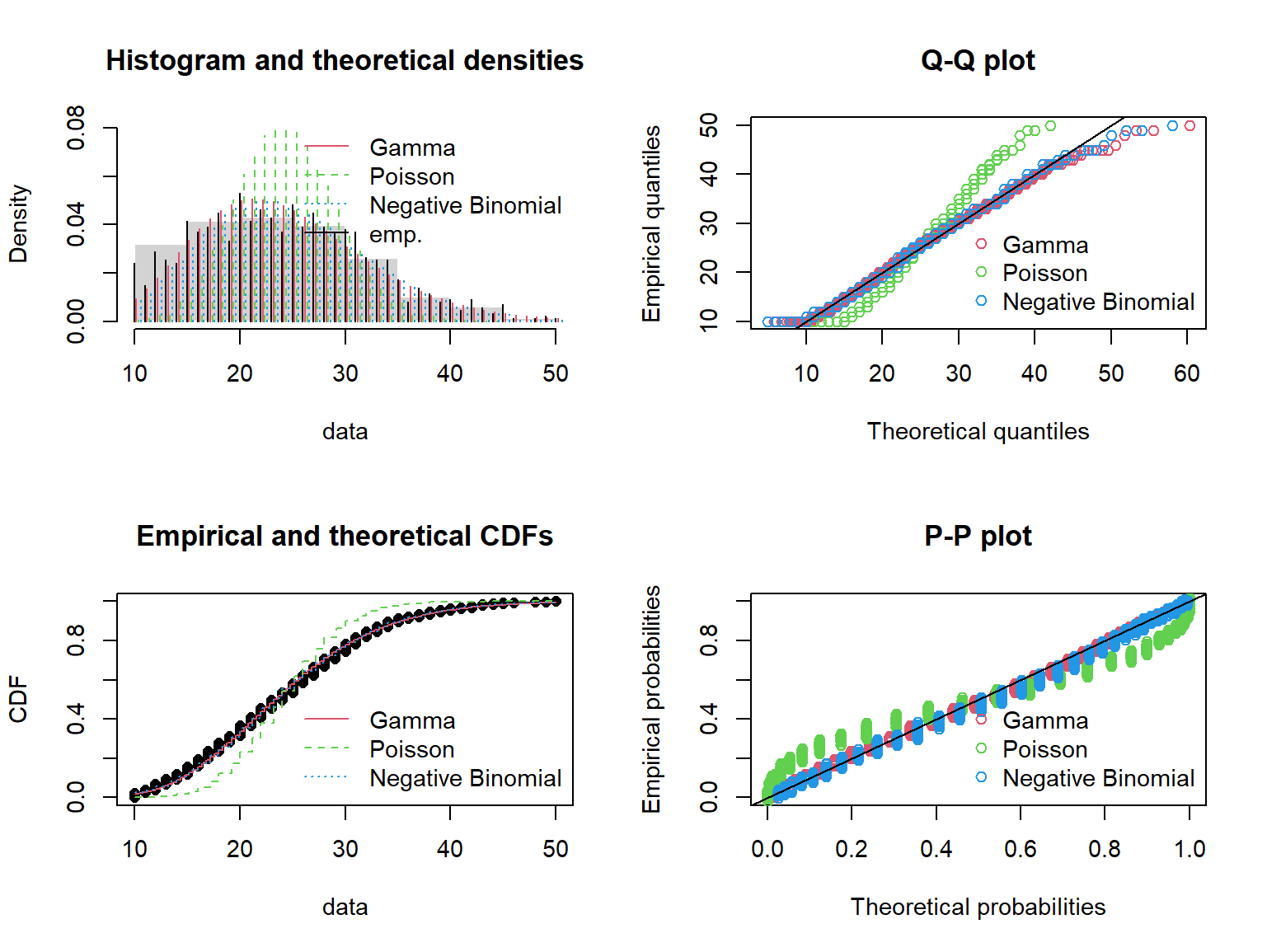


**Supplementary Figure 7. Model selection of UCLA Loneliness Scale, comparing EOD to no dementia**


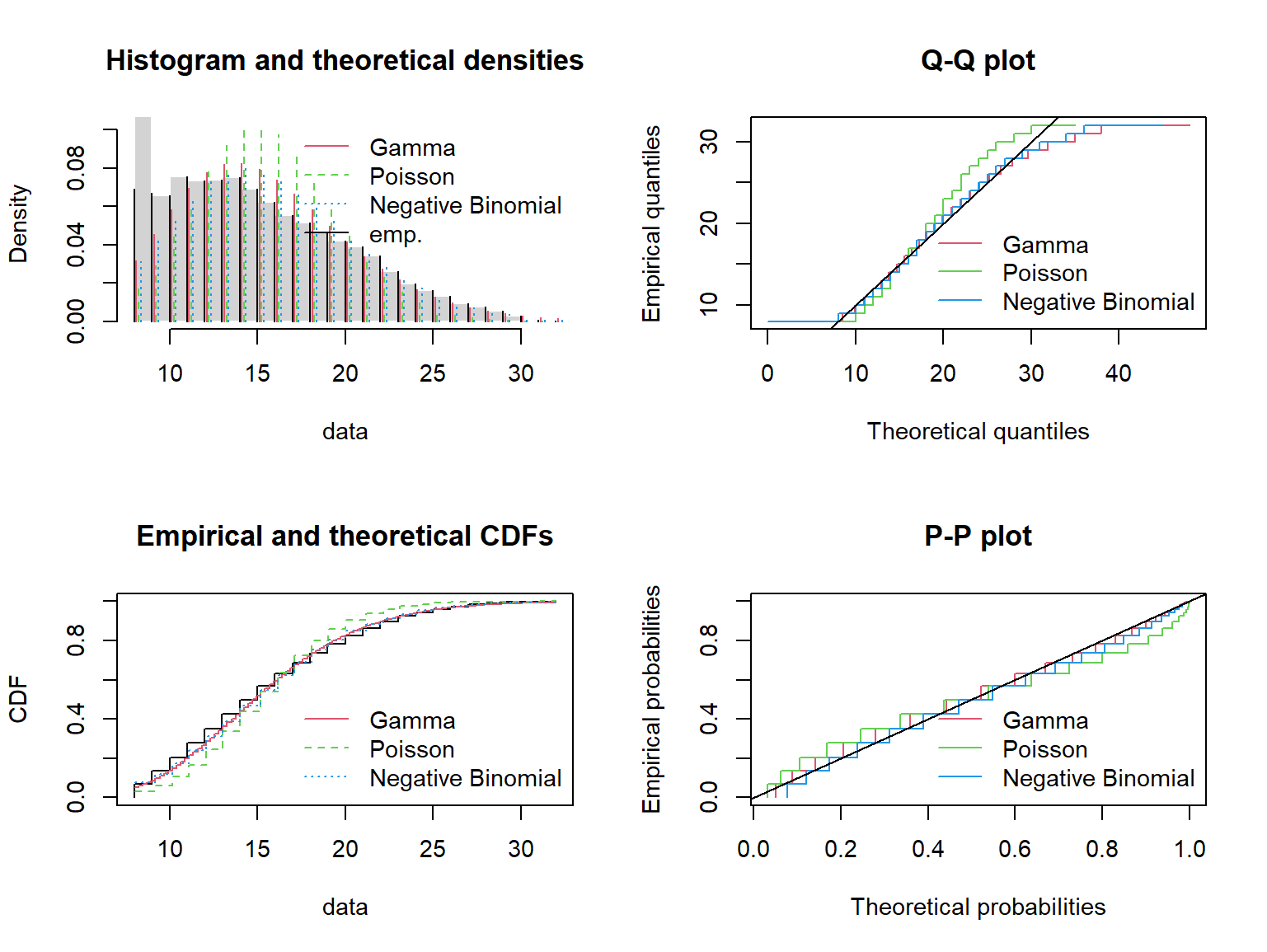


**Supplementary Figure 8. Model selection of UCLA Loneliness Scale, comparing EOD to LOD**


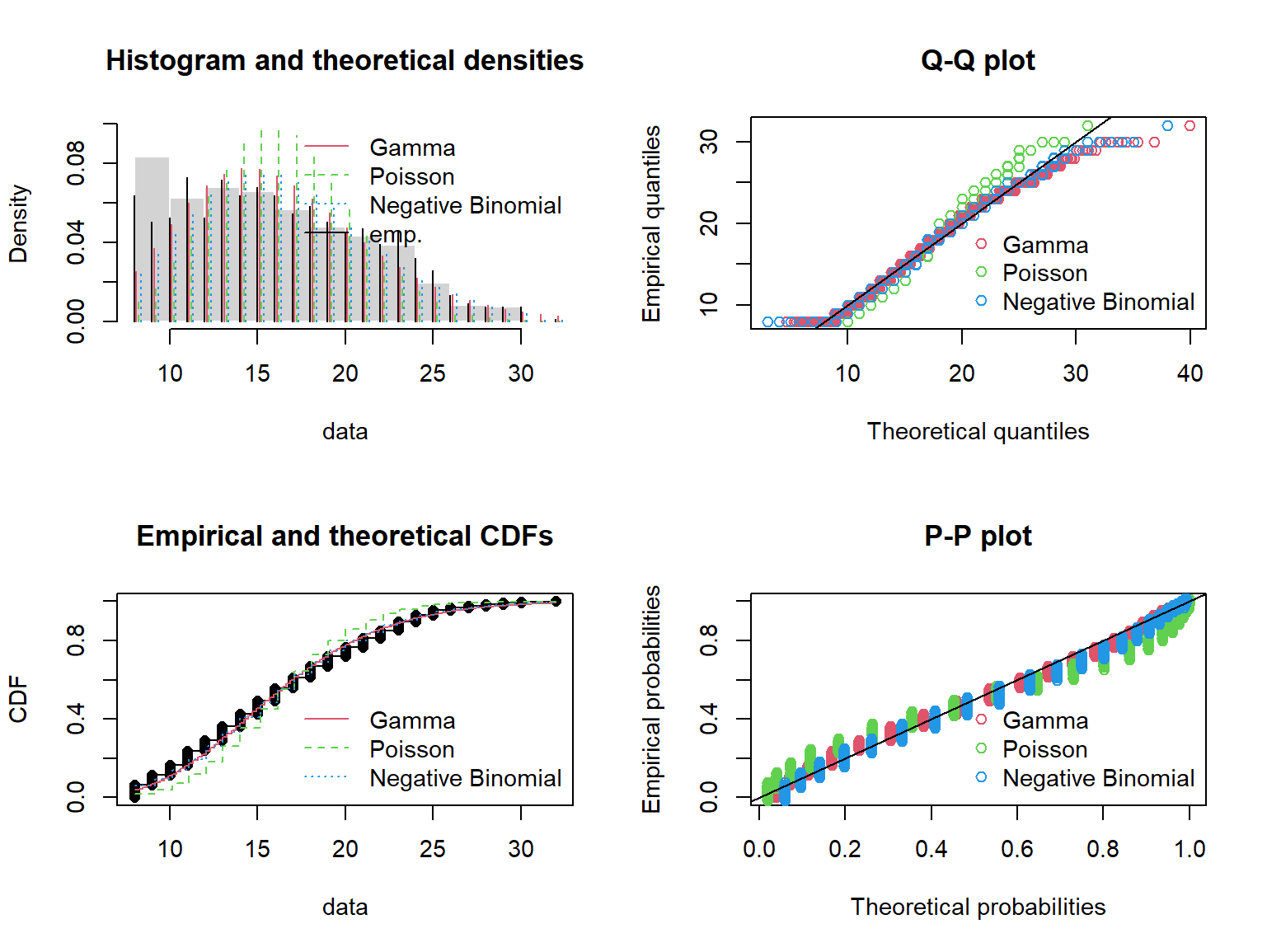

Supplement: igaf087_Supplementary_Data [file igaf087_supplementary_data.zip › innage suppl Qi, Mo, et al.docx]
